# Incidental late diagnosis of cystic fibrosis following AH1N1 influenza virus pneumonia: a case report

**DOI:** 10.1186/s13256-017-1430-0

**Published:** 2017-10-01

**Authors:** Carlo Iadevaia, Paola Iacotucci, Vincenzo Carnovale, Cecilia Calabrese, Gaetano Rea, Nicola Ferrara, Fabio Perrotta, Gennaro Mazzarella, Andrea Bianco

**Affiliations:** 1 Department of Cardio-Thoracic and Respiratory Sciences, Second University of Naples - Monaldi Hospital, Via L Bianchi, 80131 Naples, Italy; 2 Regional Adult Cystic Fibrosis Centre Department of Translational Sciences, University of Naples “Federico II”, Via Pansini, 80131 Naples, Italy; 3 Radiology Unit, A.O. dei Colli – Monaldi Hospital, Via L Bianchi, 80131 Naples, Italy

**Keywords:** Pneumonia, Respiratory failure, Cystic fibrosis

## Abstract

**Background:**

Cystic fibrosis is an autosomal recessive disorder characterized by chronic progressive multisystem involvement. AH1N1 virus infections caused classic influenza symptoms in the majority of cystic fibrosis patients while others experienced severe outcomes.

**Case presentation:**

We report a case of late incidental cystic fibrosis diagnosis in a young Caucasian man suffering from respiratory failure following infection due to AH1N1 influenza virus. The patient was admitted to our department with fever, cough, and dyspnea at rest unresponsive to antibiotics

**Conclusions:**

Late diagnosis of cystic fibrosis in uncommon. This report highlights the importance of early cystic fibrosis diagnosis to minimize risk of occurrence of potential life-threatening complications.

## Background

Cystic fibrosis (CF) is a chronic multisystem progressive disease that involves the respiratory tract, pancreas, intestine, sweat glands and reproductive tract. Worldwide incidence is 1:2500 live births with mutations carried by 1:26–27 individuals [1]. CF is a common autosomal recessive disorder caused by mutations of the cystic fibrosis transmembrane regulator gene (CFTR), classified in six subgroups on the basis of biomolecular mechanisms [1].

Improvement in diagnosis and therapy has resulted in early detection and prolonged life expectancy. CF is usually diagnosed at newborn screening (NBS) [2].

Late diagnosis (LD-CF) may occur and is being made more frequently. When CF is diagnosed in adults, it appears to be clinically different to pediatric presentation. Prognosis and life expectancy seems to be better when the disease is diagnosed in adulthood although the disease course is seriously affected by frequent respiratory infections and early bacterial colonization [2, 3].

The main cause of morbidity and mortality of CF adult patients is due to bronchial and lung involvement causing chronic bronchiectasis, which is responsible for more than 90% of fatal events. Chronic pulmonary bacterial colonization and recurrent infectious exacerbations dominate the clinical picture in CF.

Chronic pulmonary colonization is characterized by *Staphylococcus aureus* and *Haemophilus influenza* in infancy with the appearance of *Pseudomonas aeruginosa* toward the end of the first decade, and during the second and third decades the development of pathogens such as *Burkholderia cepacia, Stenotrophomonas maltophilia* and *Achromobacter* species [4, 5]*.*

Viral infections determine inflammation and cell damage within the respiratory tract. Common respiratory viral infections are associated, in CF patients, with higher risk of mortality due to rapid deterioration of pulmonary function and an increased risk of bacterial co-infections [6, 7].

A/California/7/2009 (H1N1) virus has been responsible for a worldwide influenza pandemic [8, 9]. The majority of AH1N1 virus infections cause classic influenza symptoms while a number of patients with underlying medical conditions, including chronic lung disorders such as CF, experience severe disease also due to bacterial co-infection [10, 11].

## Case presentation

In January 2014, a Caucasian 19-year-old man was admitted to the Department of Cardio-Thoracic and Respiratory Sciences at the Second University of Naples with severe respiratory failure secondary to a bilateral pneumonia.

Our patient reported 15 days of fever (with a maximum of temperature 40 °C) associated with productive cough; no improvement of symptoms with oral antibiotic therapy was achieved at home. On admission, our patient presented with a high fever of 40 °C, malaise, and dyspnea (Borg scale 2). Despite mild dyspnea, arterial blood gases (ABGs) on admission showed severe hypoxic respiratory failure with a partial pressure of arterial oxygen (PaO_2_) of 49 mmHg, partial pressure of carbon dioxide (PCO_2_) of 45 mmHg, and oxygen saturation (SpO_2_) of 90% on fraction of inspired oxygen (FiO_2_) 21%.

His family history revealed no respiratory disorders while his clinical history revealed frequent episodes of bronchitis and wheezing treated by a general practitioner with inhalers and antibiotics and never investigated by a pulmonologist.

A clinical examination exhibited diffuse rhonchi and crackles throughout both lung fields, digital clubbing, hypotonia with generalized muscular hypotrophy, and small body size for his age; his body mass index (BMI) on admission was 16.

Spirometry indicated the presence of a severe obstructive ventilatory defect.

**Fig. 1:**
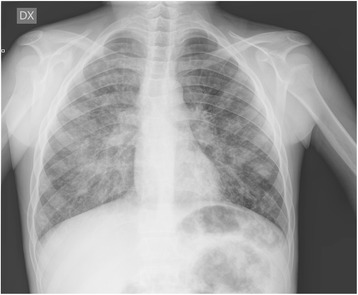
Chest X-ray: multiple bilateral opacities and reticular pattern in both thoracic fields

A chest X-ray (CXR) exhibited extensive, undefined micronodular opacities and also mild reticular lines in the middle and lower thoracic fields (Fig. 1).

Blood examinations showed normochromic normocytic anemia (Hgb (haemoglobin) 10,6 g/dL), marked leukocytosis (31.18*10^3^u/L), and hypergammaglobulinemia (IgG (immunoglobulin G) 2136 mg/dL).

AH1N1 virus infection was laboratory confirmed on pharyngeal swab by using a real-time reverse-transcriptase polymerase chain reaction assay (rapid diagnostic test for H1N1 influenza virus (FAST SET) H1N1v Arrow Diagnostic srl Genova, Italy).

Concurrent *Pseudomonas aeruginosa* and *Acinetobacter baumanii* complex infection was detected on sputum microbial examination; antibiotic therapy was administered as follows: piperacillin-tazobactam 4.5gr intravenously three times a day and ciprofloxacin 400 mg intravenously twice a day in addition to oseltamivir orally . Our patient was placed in isolation in a negative pressure room until pharyngeal swabs for AH1N1 were clear.

Despite pharmacological therapy and oxygen support, his clinical state remained poor. His PO_2_ value was 60 mmHg and PCO_2_ 44 mmHg on oxygen via a 40% venturi mask.

**Fig. 2:**
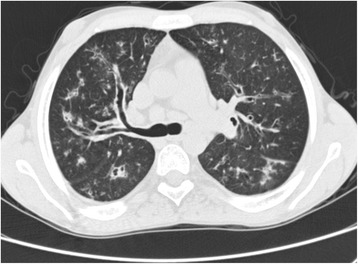
High-resolution computed tomography: cylindrical bronchiectasis, mucous plugging, and centrilobular nodules, a tree-in-bud pattern

A high-resolution computed tomography (HRCT) scan identified multiple cylindrical bronchiectasis and bronchiolectasis in both lungs as well as mucous plugging and centrilobular nodules. Also evident in some areas were “tree-in-bud opacities”, an expression of acute small airways disease. An incidental finding was adipose infiltration of the pancreas (Fig. 2).

At this stage clinical and radiological findings raised suspicion of CF; therefore, our patient underwent a sweat test, which exhibited clear positive values (a Na+ of 103 mEq/L and Cl- 94 mEq/L) and a molecular test with deoxyribonucleic acid (DNA) extraction and analysis of CFTR gene mutations confirming disease (genotype DeltaF508/DeltaF508).

Our patient was then transferred to the Adult Cystic Fibrosis Department of Federico II Hospital, University of Naples; a clinical examination showed the presence of dyspnea (Borg scale 3), fever (a temperature of 39 °C), asthenia, cachectic appearance, immobilization syndrome, presence of diffuse rhonchi, and crackles throughout both lung fields as well as a sacral pressure sore (stage 1).

ABGs on oxygen therapy with a nasal cannula (4 L/min) showed values of PCO_2_ of 34.4 mmHg and PO_2_ of 58.1 mmHg.

Blood examinations confirmed anemia (hemoglobin 8.8 g/dL), marked leukocytosis 20,000/L, hypergammaglobulinemia and C- reactive protein (PCR) of 5.14 mg/dL; persistence of *Pseudomonas aeruginosa* and *Acinetobacter baumanni* complex was showed on sputum. Parenteral antibiotic therapy was continued as follows: meropenem 2 g intravenously three times a day, ceftazidime 3 g intravenously three times a day, colistin 2,000,000 intravenously three times a day. Further therapy included acetylcysteine intravenously, twice a day, electrolytic 0.9% solution, intravenously, betamethasone 1.5 mg, intravenously, twice a day.

A program of drainage physiotherapy was started using ELTGOL (slow expiration with glottis opened in lateral posture, French: Expiration Lente Totale Glotte Ouverte en décubitus Latéral), positive expiratory pressure (PEP) mask, Acapella and autogenous drainage twice a day. To improve his bronchial hygiene and physiotherapy, nebulized hypertonic saline 3% solution was administered.

An air mattress was used to aid healing of a sacral pressure sore and low molecular weight heparin was administered while our patient was immobile. Parenteral ferrous sulfate was prescribed to treat anemia. Dietary assessments were carried out during admission to optimize nutritional intake.

His pancreatic insufficiency was treated with pancreolipase and the diabetes was well controlled with rapid-acting insulin three times daily and long-acting insulin therapy overnight.

During the 3 months’ admission, our patient demonstrated significant improvement in his respiratory symptoms; normalization of arterial blood gas values, good glycemic control, satisfactory weight gain (BMI 18), resolution of his sacral pressure sore, and improvement in his energy levels leading to remobilization [12].

Our patient was discharged to attend rehabilitative therapy as an outpatient (Table 1).

**Table 1:** Clinical and functional data of our patient at 1-year follow-up

	T0	T1	T3	T6	T12
FEV 1	29%	30%	38%	40%	40%
BMI	16	16	18	19	19
SpO_2_	90%	90%	93%	95%	95%
Number of bronchial exacerbation	/	0	0	1	1

*T0* time of admission, *T1* 1 month later diagnosis, *T3* 3 month later diagnosis, *T6* 6 month later diagnosis, *T12* 12 month later diagnosis, *FEV 1* forced expiratory volume in the 1st second, *BMI* body mass index, *SPO*_*2*_ peripheral capillary oxygen saturation

## Discussion

Although CF is usually discovered early in life, it is no longer only a pediatric disease.

As is reported in the literature, patients who receive early diagnosis of CF suffer from a combination of gastrointestinal and pulmonary symptoms, while respiratory disorders are more common when diagnosis is late. In adulthood, CF may be diagnosed on the basis of isolated symptoms ranging from male infertility to episodes of recurrent pancreatitis [3]; other patients have mild symptoms mimicking other respiratory disorders [13, 14] with absence of digestive symptoms.

In our case, despite extensive lung involvement, late diagnosis of CF was incidentally performed following severe respiratory infection due to AH1N1 influenza virus. Advances in diagnostic procedures, including imaging [15, 16], have contributed to early cystic fibrosis suspicion on admission. Late diagnosis (LD-CF) is possible and is defined as an individual who fulfils the criteria for CF and is either NBS-negative (LD-NBS-neg) with a negative immunoreactive trypsinogen (IRT) or genotype, or NBS-positive but with a sweat chloride (SC) < 60 mmol/L [3, 17].

The sweat test of Gibson and Cooke remains the most sensitive and cheapest method for CF diagnosis when carried out in high specialization centers. In Italy, CF diagnosis is performed using a neonatal screening procedure; this minimizes the occurrence of late diagnosis.

Chronic respiratory disorders arising from different pathways [18–21] have been linked to severe lung involvement leading to respiratory deterioration, requiring oxygen support or mechanical ventilation and even death in AH1N1 virus infection.

Viral infections seems to have the same occurrence between CF and healthy subjects but patients suffering from CF are more frequently symptomatic and the course of the disease worse. The role of pandemic A (H1N1) virus infection in CF patients is unclear, but these patients seem to be more susceptible to the infection. Severe respiratory involvement is an important risk factor for intensive care unit (ICU) admission and death [22–24].

## Conclusions

The widespread availability of centers for the analysis of CFTR and the discovery of new drugs modulating the chloride protein channel have resulted in a raised awareness of cystic fibrosis diagnosis [1, 17] in adult patients also.

The diagnosis should be considered even when nontypical symptoms are exhibited. Missed CF diagnosis and treatment has been responsible, in our case, for a severe and potential life-threatening respiratory complication caused by AH1N1 influenza virus infection.

It is important to reduce the occurrence of late CF diagnosis and promote appropriate patient care.

## Abbreviations

ABG: Arterial blood gases
CF: Cystic fibrosis
CFTR: Cystic fibrosis transmembrane regulator gene
CXR: Chest X ray
HRCT: High-resolution computed tomography
LD-CF: Late-diagnosis cystic fibrosis
LD-NBS-neg: Late diagnosis – negative newborn screening

## References

[1] Rowe SM, Miller S, Sorscher EJ. Cystic fibrosis. N Engl J Med. 2005;352:1992–2001. 10.1056/NEJMra043184 15888700

[2] Coffey MJ, Whitaker V, Gentin N, Junek R, Shalhoub C, Nightingale S, Hilton J, Wiley V, Wilcken B, Gaskin KJ, Ooi CY. Differences in outcomes between early and late diagnosis of cystic fibrosis in the newborn screening era. J Pediatr. 2017;181:137–45. e1. 10.1016/j.jpeds.2016.10.045 27837951

[3] Santos V, Cardoso AV, Lopes C, Azevedo P, Gamboa F, Amorim A. Cystic fibrosis - Comparison between patients in paediatric and adult age. Rev Port Pneumol. 2017;23(1):17–21. 10.1016/j.rppnen.2016.07.002 27743767

[4] Rosenstein BJ, Zeitlin PL. Cystic fibrosis. Lancet. 1998;351:277–82. 10.1016/S0140-6736(97)09174-5 9457113

[5] Laurans M, Arion A, Fines-Guyon M, Regeasse A, Brouard J, Leclercq R, Duhamel JF. Pseudomonas aeruginosa and cystic fibrosis: first colonization to chronic infection. Arch Pediatr. 2006;13(Suppl1):S22–9. 17370393

[6] Whiteman SC, Bianco A, Knight RA, Spiteri MA. Human rhinovirus selectively modulates membranous and soluble forms of its intercellular adhesion molecule-1 (ICAM-1) receptor to promote epithelial cell infectivity. J Biol Chem. 2003;278:11954–961. 10.1074/jbc.M205329200 12551926

[7] Bianco A, Sethi SK, Allen JT, Knight RA, Spiteri MA. Th2 cytokines exert a dominant influence on epithelial cell expression of the major group human rhinovirus receptor, ICAM-1. Eur Respir J. 1998;12:619–26. 10.1183/09031936.98.12030619 9762790

[8] Viviani L, Assael BM, Kerem E. Impact of the A (H1N1) pandemic influenza (season 2009-2010) on patients with cystic fibrosis. J Cyst Fibros. 2011;10:370–6. 10.1016/j.jcf.2011.06.004 21752728

[9] Writing Committee of the WHO Consultation on Clinical Aspects of Pandemic (H1N1) 2009 Influenza, Bautista E, Chotpitayasunondh T, Gao Z, Harper SA, Shaw M, Uyeki TM, Zaki SR, Hayden FG, Hui DS, Kettner JD, Kumar A, Lim M, Shindo N, Penn C, Nicholson KG. Clinical aspects of pandemic 2009 influenza A (H1N1) virus infection. N Engl J Med. 2010;362(18):1708–19. 10.1056/NEJMra1000449 20445182

[10] Bianco A, Parrella R, Esposito V, Mazzarella G, Sammarco ML, Brunese L, Ripabelli G. Severe A(H1N1)-associated pneumonia sequential to Clamidophila pneumoniae infection in Healthy Subject. In Vivo. 2011;25(5):825–8. 21753141

[11] Giannattasio A, Brunese L, Ripabelli G, Mazzarella G, Bianco A. Coinfections with influenza virus and atypical bacteria: implications for severe outcomes? Clin Respir J. 2016. [Epub ahead of print]. 10.1111/crj.12510 27249224

[12] De Simone G, Aquino G, Di Gioia C, Mazzarella G, Bianco A, Calcagno G. Efficacy of aerobic physical retraining in a case of combined pulmonary fibrosis and emphysema syndrome: a case report. J Med Case Rep. 2015;9:85. 10.1186/s13256-015-0570-3 PMC4422471 25928714

[13] Mazzarella G, Iadevaia C, Guerra G, Rocca A, Corcione N, Rossi G, Amore D, Brunese L, Bianco A. Intralobar pulmonary sequestration in an adult female patient mimicking asthma: a case report. Int J Surg. 2014;12 Suppl 2:S73–7. 10.1016/j.ijsu.2014.08.376 25159547

[14] Couriel J. Assessment of the child with recurrent chest infections. Br Med Bull. 2002;61:115–32. 10.1093/bmb/61.1.115 11997302

[15] Maniscalco M, Bianco A, Mazzarella G, Motta A. Recent advances on nitric oxide in the upper airways. Curr Med Chem. 2016;23(24):2736–45. 10.2174/0929867323666160627115335 27356533

[16] Brunese L, Greco B, Setola FR, Lassandro F, Guarracino MR, De Rimini M, Piccolo S, De Rosa N, Muto R, Bianco A, Muto P, Grassi R, Rotondo A. Non-small cell lung cancer evaluated with quantitative contrast-enhanced CT and PET-CT: net enhancement and standardized uptake values are related to tumour size and histology. Med Sci Monit. 2013;19:95–101. 10.12659/MSM.883759 PMC3628799 23396358

[17] Bombieri C, Seia M, Castellani C. Genotypes and phenotypes in cystic fibrosis and cystic fibrosis transmembrane regulator-related disorders. Semin Respir Crit Care Med. 2015;36:180–93.16. 10.1055/s-0035-1547318 25826586

[18] Bianco A, Mazzarella G, Turchiarelli V, Nigro E, Corbi G, Scudiero O, Sofia M, Daniele A. Adiponectin: an attractive marker for metabolic disorders in chronic obstructive pulmonary disease (COPD). Nutrients. 2013;5(10):4115–25. 10.3390/nu5104115 PMC3820062 24128974

[19] Bianco A, Nigro E, Monaco ML, Matera MG, Scudiero O, Mazzarella G, Daniele A. The burden of obesity in asthma and COPD: role of adiponectin. Pulm Pharmacol Ther. 2017;43:20–5. 10.1016/j.pupt.2017.01.004 28115224

[20] Nigro E, Daniele A, Scudiero O, Ludovica Monaco M, Roviezzo F, D'Agostino B, Mazzarella G, Bianco A. Adiponectin in asthma: implications for phenotyping. Curr Protein Pept Sci. 2015;16(3):182–7. 10.2174/1389203716666150120095342 25760347

[21] Nigro E, Scudiero O, Sarnataro D, Mazzarella G, Sofia M, Bianco A, Daniele A. Adiponectin affects lung epithelial A549 cell viability counteracting TNFα and IL-1ß toxicity through AdipoR1. Int J Biochem Cell Biol. 2013;45(6):1145–53. 10.1016/j.biocel.2013.03.003 23500159

[22] Renk H, Regamey N, Hartl D. Influenza A(H1N1)pdm09 and cystic fibrosis lung disease: a systematic meta-analysis. PLoS One. 2014;9(3):e93142. 10.1371/journal.pone.0078583 PMC3888399 24427261

[23] Colombo C, Battezzati PM, Lucidi V, Magazzu G, Motta V, *et al.* Influenza A/H1N1 in patients with cystic fibrosis in Italy: a multicentre cohort study. Thorax. 2011;66:260–1. 10.1136/thx.2010.157032 21228422

[24] France MW, Tai S, Masel PJ, Moore VL, McMahon TL, *et al.* The month of July: an early experience with pandemic influenza A (H1N1) in adults with cystic fibrosis. BMC Pulm Med. 2010;10:8. 10.1186/1471-2466-10-8 PMC2837636 20181286

